# Genomic characteristics of *Mycobacterium tuberculosis* isolates of cutaneous tuberculosis

**DOI:** 10.3389/fmicb.2023.1165916

**Published:** 2023-05-17

**Authors:** You-Ming Mei, Wen-Yue Zhang, Ji-Ya Sun, Hai-Qin Jiang, Ying Shi, Jing-Shu Xiong, Le Wang, Yan-Qing Chen, Si-Yu Long, Chun Pan, Tao Luo, Hong-Sheng Wang

**Affiliations:** ^1^Hospital of Skin Disease, Institute of Dermatology, Chinese Academy of Medical Sciences and Peking Union Medical College, Nanjing, China; ^2^Jiangsu Key Laboratory of Molecular Biology for Skin Diseases and STIs, Nanjing, China; ^3^Center for Systems Medicine, Institute of Basic Medical Sciences, Chinese Academy of Medical Sciences & Peking Union Medical College, Suzhou, China; ^4^Suzhou Institute of Systems Medicine, Suzhou, China; ^5^Department of Pathogen Biology, West China School of Basic Medical Sciences and Forensic Medicine, Sichuan University, Chengdu, China

**Keywords:** mycobacteria infection, *Mycobacterium tuberculosis*, whole genome sequence, cutaneous tuberculosis, comparative genomics

## Abstract

**Objectives:**

Cutaneous tuberculosis with various manifestations can be divided into several clinical types according to the host's immune status and infective route. However, the etiological factors of this disease remain unclear. The objective of this study is to investigate the pathogens associated with the occurrence and different types of cutaneous tuberculosis.

**Methods:**

58 *Mycobacterium tuberculosis* strains isolated from cutaneous tuberculosis over the last 20 years were sequenced and analyzed for genomic characteristics including lineage distribution, drug-resistance mutations, and mutations potentially associated with different sites of infection.

**Results:**

The *M. tuberculosis* strains from four major types of cutaneous tuberculosis and pulmonary tuberculosis shared similar genotypes and genomic composition. The strains isolated from cutaneous tuberculosis had a lower rate of drug resistance. Phylogenic analysis showed cutaneous tuberculosis and pulmonary tuberculosis isolates scattered on the three. Several SNPs in metabolism related genes exhibited a strong correlation with different infection sites.

**Conclusions:**

The different infection sites of TB may barely be affected by large genomic changes in *M. tuberculosis* isolates, but the significant difference in SNPs of drug resistance gene and metabolism-related genes still deserves more attention.

## Introduction

The *Mycobacterium tuberculosis* complex consists of several groups with geographic origins and distributions. Among them, *M. tuberculosis sensu stricto* (MTB, Lineages 1, 2, 3, 4, and 7) and *M. tuberculosis var. africanum* (Lineages 5 and 6) are the major pathogens of human disease. Tuberculosis, which is caused by infection with the *M. tuberculosis* complex, remains a threat to global public health (World Health Organization, 2021). It can be simply divided into pulmonary tuberculosis (PTB) and extrapulmonary tuberculosis (EPTB). EPTB is less contagious and has received secondary attention in most tuberculosis surveillance programs, but research has revealed an increased proportion of this disease globally (Sandgren et al., 2013; Gomes et al., 2014). Cutaneous tuberculosis (CTB) is one of the rare forms of EPTB, and it accounts for only 1–2% of tuberculosis cases (Franco-Paredes et al., 2018). CTB is conventionally classified into several groups with characteristic manifestations. The various clinical forms were previously supposed to be the result of both different infective routes and host immune statuses. Lupus vulgaris (LV), tuberculosis cutis orificialis (TCO), and scrofuloderma (SFD) are considered to be disseminators of visceral tuberculosis, while tuberculosis verrucosa cutis (TVC) and tuberculosis chancre are supposed to develop through exogenous inoculation (Hill and Sanders, 2017). However, the current classification rule is mainly derived from clinical inference. The temperature is an important factor for the growth of the microorganism. Mycobacteria such as *M. marinum* show comparatively strict temperature limits in human infection. In general, the skin provides a more stressful environment than other visceral organs, including the lower temperature and hypoxia, which are unfit for MTB infection and proliferation, which may suggest the special fitness of these CTB isolates (Jabir et al., 2017). However, we found few studies focusing on the potential etiological factors of CTB or reporting the characteristics of CTB isolates.

Approaches based on whole genome sequencing provide superior insights into mutation-based mycobacterial genotyping, drug resistance profiling, and transmission detection. Analysis models based on comparative genomics have successfully identified several novel resistance or adaptation-related mutations in MTB (Desjardins et al., 2016; Farhat et al., 2019). Many studies discuss MTB isolates from pulmonary sites or other extrapulmonary sites, but the genome sequence of the CTB isolate has not been reported to date (Sandgren et al., 2013; Gomes et al., 2014; Desjardins et al., 2016). Thus, this study collected CTB isolates in China to investigate the pathogen genomic characteristics of CTB.

## Methods

### Sample collection

We selected 111 cases suspected as CTB confirmed by PCR amplification and sequencing for *16S, rpoB*, or *hsp65* genes of skin biopsy, or by sequencing of tissue cultures in the Hospital of Dermatology, Chinese Academy of Medical Sciences from 2000 to 2020. All the clinical records for these cases were retrospectively reviewed. The patients' skin biopsy was collected before anti-TB therapy and ground and spread on improved Lowenstein–Jensen slants at 32 and 37°C, respectively. The clinical culture time till visible colonies appeared in 87 strains was recorded (Table 1). The remaining organisms after identification were routinely freeze-preserved at −80°C. In 2021, all the existing frozen stocks (73) were sub-cultured at 37°C for 4–12 weeks to acquire enough organisms for sequencing. The stored strains unsuccessfully recovered or had contamination were excluded in the following study. Finally, 58 MTB strains isolated from CTB lesions were included in this study.

**Table 1:** Clinical information of cutaneous tuberculosis and the growth temperature of these isolates.

		**LV**	**TVC**	**TCO**	**SFD**	**Total**
Case		56 (64.37)	15 (17.24%)	11 (12.64)	5 (5.75%)	87
Gender (F/M)		24/32	12/3	11/0	1/4	62/49
Age		20–84	6–86	38–71	51–77	6–86
Mean age		53.09	52.57	55.00	67.00	53.46
PTB history		5 (8.93%)	0 (0%)	3 (27.27%)	1 (20%)	9 (10.34%)
Growth temperature	32°C	6 (10.71%)	3 (20%)	3 (27.27%)	0 (0%)	12 (13.79%)
		37°C	35 (62.5%)	6 (40%)	7 (63.64%)	4 (80%)	52 (59.77%)
		32/37°C	3 (5.36%)	2 (13.33%)	1 (9.09%)	0 (0%)	6 (6.9%)
		NA	12	4	0	1	17
Drug-resistant mutation		3 (5.36%)	2 (13.33%)	0 (0%)	0 (0%)	5 (5.75%)
Lineage	Lineage 2	26 (46.43%)	7 (46.67%)	4 (36.36%)	2 (40)%	39 (44.83%)
		Lineage 4	8 (14.29%)	3 (20%)	2 (18.18%)	0 (0%)	13 (14.94%)

### Whole genome sequencing and SNP calling

The organisms were lysed with 20 mg/ml lysozyme for cell wall disruption, and then, DNA was extracted using a QIAamp DNA Blood Mini Kit (Qiagen, Manchester, UK). DNA libraries with lengths of 150 bp were generated and sequenced on an Illumina HiSeq 4000 platform (Illumina, San Diego, CA, USA) at the Beijing Genomics Institute. The sequenced reads were assembled using SOAPdenovo with the reference of MTB H37Rv (Luo et al., 2012). Four strains from different CTB clinical types were sequenced using PacBio RS II and an Illumina HiSeq 4000 platform. PacBio subreads with a length of < 1 kb were removed. Draft unitigs were assembled using Celera Assembler. The non-coding RNA was detected using tRNAscan-SE, RNAmmer, and the Rfam database (Schattner et al., 2005; Karin et al., 2007; Kalvari et al., 2018). Prophage regions were predicted using PhiSpy, and CRISPR was detected by CRISPRCasFinder (Akhter et al., 2012; Couvin et al., 2018). Rapid Annotation using Subsystem Technology (RAST) was used for gene annotation (Aziz et al., 2008). All sequence data associated with this study are deposited in the NCBI Bioproject, accession PRJNA820632.

In searching for whole genome sequencing samples of MTB from southeastern China in NCBI and EMBL databases, we found 219 strains from Shanghai in the same period (PRJNA417753) (Yang C. et al., 2018). The adapters and raw reads of CTB and PTB isolates with low quality (>40% bases with base quality of < 20 or N base of >5) were trimmed using Fastp and were checked by FastQC and MultiQC for read quality (Ewels et al., 2016; Chen et al., 2018). The data sets were aligned to the reference genome sequence of *Mycobacterium tuberculosis* H37Rv (Genbank accession: GCF_000195955.2) using BWA mem (Li and Durbin, 2009). Duplicated reads were removed by Sambamba (Tarasov et al., 2015). Genomes with a read depth of < 50 × or a coverage of < 95% were excluded. Finally, 188 PTB isolates collected in 2008–2015 were selected as the group of PTB isolates.

### Bioinformatic analysis

The molecular drug resistance to TB drugs and the lineage distribution of the genomes were examined on TB-profiler trimmed data sets (Phelan et al., 2019). Roary was used to create a pan-genome, and Scoary was used to analyze the accessory gene presence or absence in CTB isolates with TCO or TVC (Page et al., 2015; Brynildsrud et al., 2016). Orthovenn2 was used for the comparative analysis of whole-genome orthologous clusters of the four genomes, representing the four CTB types (Xu et al., 2019). Descriptive statistics were used to describe the characteristics of the isolates. Chi-square tests and 95% confidence intervals (CIs) were used to compare non-metrological data by SPSS version 25 (SPSS Inc., Illinois, USA). For all analyses, a *p*-value of < 0.05 was considered to be statistically significant.

### Genome-wide association and convergence test

For the phylogeny construction and convergence test, variant calling was performed on the whole genome alignment of all the CTB and PTB strains by Snippy (Seemann, 2023). A total of 8,271 highly credible SNPs used for phylogeny construction were obtained after excluding the regions of the drug target gene, PE/PPE family, and genes with missing sites in >10% of samples. The phylogenetic tree was reconstructed using IQ-TREE assuming the GTR+F model with 1,000 bootstrap replicates, and annotation and visualization were processed by iTOL (Letunic and Bork, 2011; Nguyen et al., 2015). The convergence test was performed using the concatenated alignment of the SNP site of the 58 CTB isolates and the tree above (Page et al., 2016). The homoplastic mutations independently occurring in different clades were found in the particular site with the top frequency of change along the tree with tree-time (Sagulenko et al., 2018). GWAS analyses were performed on non-synonymous SNPs in CDS using the burden test in PYSEER (Lees et al., 2018). Pairwise distances calculated from the phylogeny tree were used to account for relatedness. A *P*-value threshold of < 1E-04 was considered to be statistically significant. The products and the likely impact of the gene with SNP were annotated by SnpEff (Cingolani et al., 2012). The published algorithm was used to predict the effects of identified mutations on protein stability (Capriotti et al., 2005).

## Results

### Clinical information and laboratory features of CTB isolates

A total of 111 HIV-negative cases with a positive culture and conserved gene sequencing results of MTB from 2000 to 2020 were reviewed in this study. Among the cases with intact geographic information, most of them came from Jiangsu (53.47%, 54/101) and Anhui provinces (26.73%, 27/101) (Table 1). The average age of all patients was 53.46 ± 16.26 years (range from 6 to 86 years, median of 54), and 55.86% (62/111) were male patients. A total of 87 cases could be categorized into four major CTB classifications based on medical records, images, and pathological examination results, and nine patients (10.34%) had signs of active infection or a history of visceral tuberculosis. LV was the major form of CTB (64.37%, 56/87), followed by TVC (15/87, 17.24%) and TCO (11/87, 12.64%), which is similar to previous literature reports in China (Zhang et al., 2018). Among the 45 patients with intact treatment records, 93.33% (42/45) of patients recovered well after 6 months of standard multidrug therapy. Three cases recurred after anti-tuberculosis therapy more than 10 years later.

### Genomic characteristics of CTB isolates

A total of 58 CTB isolates were successfully recovered, were sequenced on the Illumina HiSeq 4000 platform, and were assembled reads using SOAPdenovo with the reference of MTB H37Rv (Methods). After all the CTB isolates were examined on TB-profiler trimmed reads, these CTB isolates were confirmed as MTB with no isolates of other subspecies, such as *M. bovis*, found. Lineage 2.2 (Beijing family strains) predominated in all cases (41/58, 70.69%). The remaining 17 isolates belonged to Lineage 4, in which Lineage 4.4 was the most prevalent sub-lineage (8/17, 47.06%), followed by Lineages 4.2 and 4.5 (4/17, 23.53%, respectively). No strains of Lineage 1 or Lineage 3 were found in these specimens. The lineage, growth time, or temperature of strains of different CTB types revealed no significant differences. Seven CTB strains were found that had resistance mutations, and they accounted for only 12.07% of all the CTB isolates (Tables 2, 3). Most of these isolates were mono-drug resistant (4/7, 57.14%), and they were mainly resistant to isoniazid or streptomycin. The only MDR isolate detected with resistance to rifampicin, isoniazid, ethambutol, and streptomycin was collected from a recurrent LV after 20 years of treatment. A higher proportion of drug resistance was observed in Lineage 2.2 strains (14.63%, 6/41) than in Lineage 4 strains (5.88%, 1/17), with no significant difference (χ^2^ = 0.239, *p* = 0.625).

**Table 2:** Drug resistance mutations in cutaneous tuberculosis isolates.

**Drug**	**Mutation**	**Strains**
RFP	rpoB_p.Ser450Leu	1
INH	katG_p.Ser315Thr	4
EMB	embB_p.Met306Ile	1
STR	rpsL_p.Lys88Arg	3
	gid_c.115_115del	1
PAS	thyA_p.His75Asn	2
**DR type**	**Drug**	**Strains**
Drug resistant	INH	1
	STR	1
	PAS	2
	INH + STR	2
MDR	RFP + INH + EMB + STR	1

**Table 3:** Drug resistance strain distribution in isolates of cutaneous tuberculosis and pulmonary tuberculosis in adjacent provinces.

	**CTB**	**PTB**	
**DR**	***n*** = **58 (Jiangsu, 2000–2019)**	***n*** = **260, (Jiangsu, 2010)** **(Liu et al., 2011)**	***n*** = **2,133, (Anhui, 2015–2016)** **(Yao et al., 2020)**
RFP	1 (1.72%)	38 (14.62%)	163 (7.64%)
INH	4 (6.90%)	45 (17.31%)	223 (10.45%)
EMB	1 (1.72%)	20 (7.69%)	40 (1.88%)
STR	4 (6.90%)	43 (16.54%)	234 (10.97%)
Mono-DR	4 (6.90%)	33 (12.7%)	-
MDR	1 (1.72%)	34 (13.1%)	106 (4.97%)
XDR	-	-	13 (0.61%)
Total	7 (12.07%)	74 (28.5%)	-

**Figure 1:**
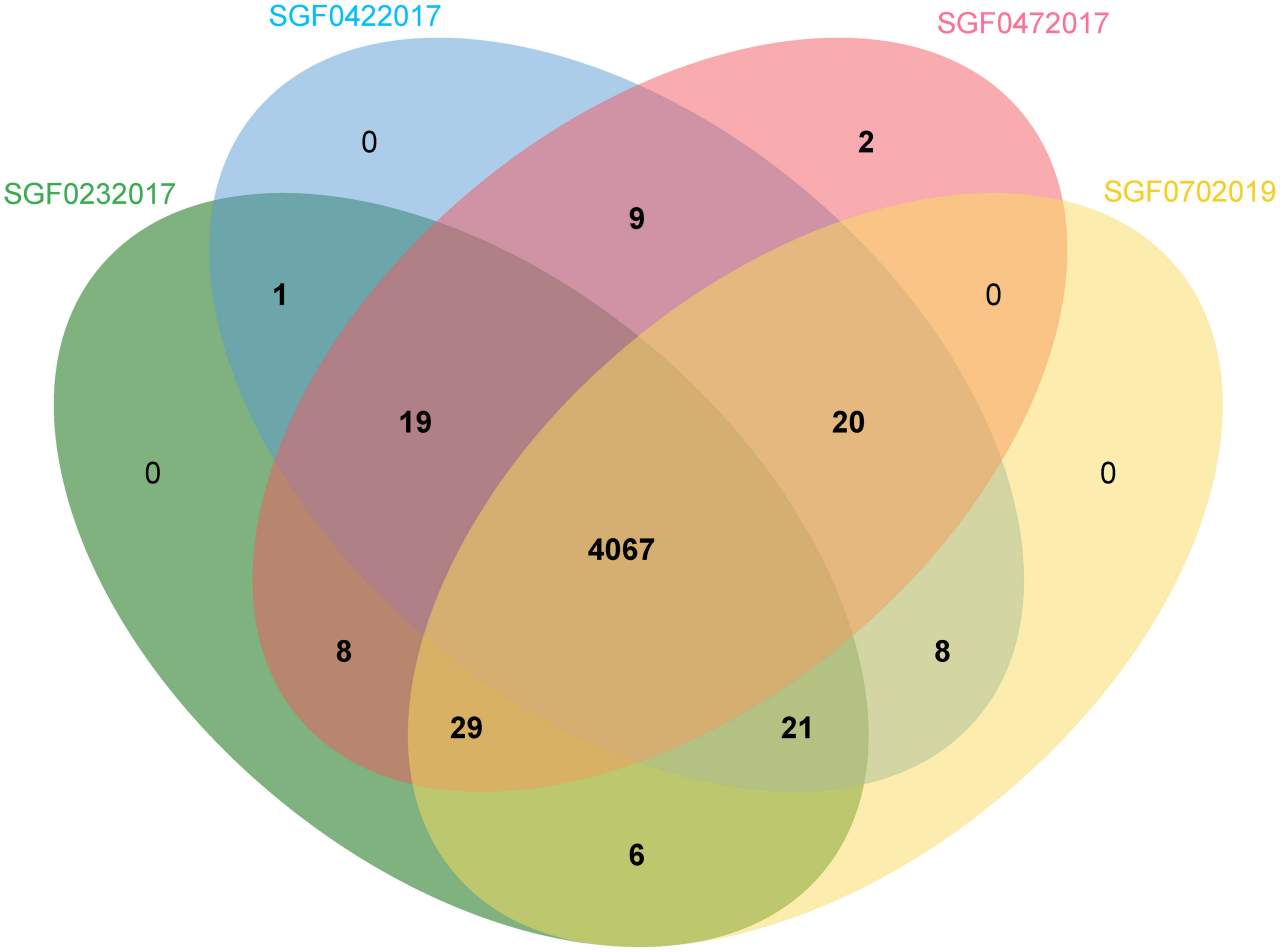
Venn image of the orthologous genes of four representative genomes from different CTB types.

The covered length of the draft CTB genomes ranges from 4,348,399 to 4,406,327 bp (98.57–99.88%) compared with the reference MTB H37Rv (Supplementary Table 1). These genomes have a GC content between 64.10 and 65.61%. In the pan-genome analysis, 5,219 orthologous genes including 3,518 core genes and other variable components (Softcore: 247, Cloud: 1,032, Shell: 422) were identified. The pan-genome curve indicated an open pan-genome of MTB as previously reported (Yang C. et al., 2018). In a comparison of TVC and TCO, which were the specific CTB types caused by exogenous and endogenous infections, respectively, we found that the existence of several genes significantly differed in isolates of TVC and TCO, including *PPE47* (6/6 in TVC, 4/10 in TCO), *embR* (3/6 in TVC, 10/10 in TCO), and *moaA1* (3/6 in TVC, 10/10 in TCO), as well as three coding genes of putative proteins. However, these differences need to be further confirmed because the technical errors in short-read sequencing may not be totally eliminated. We performed PacBio genome sequencing in four representative Lineage 2 strains to acquire more precise *de novo* assemblies of different CTB groups to thoroughly investigate the genome of isolates from different CTB types. The basic information of the four assemblies is summarized in Table 4. A total of 4,286–4,303 protein-coding genes and 59 non-coding RNAs including 45 tRNAs, 3 rRNAs, and 15 sRNAs were annotated. The orthologous gene clusters showed similar compositions of these strains, and only the isolate of SFD was annotated with an additional unique gene of the transposase of the IS1081 element (Figure 1).

**Table 4:** Genomic information of the four cutaneous tuberculosis isolates from different clinical types.

	**SGF0232017**	**SGF0422017**	**SGF0472017**	**SGF0702019**	***M. tuberculosis* H37Rv**
Clinical type	TVC	LV	SGD	TVC	
Length	4,413,076	4,402,590	4,417,237	4,419,329	4,411,532
GC (%)	65.60	65.61	65.61	65.62	65.6
Coding sequences	4,298	4,286	4,330	4,303	4,299
Subsystems	293	293	293	292	294
Number of prophage	5	6	10	5	5
Number of CRISPR	8	8	8	9	9
tRNA (% in genome)	45	45	45	45	45
5s_rRNA (% in genome)	1	1	1	1	1
16s_rRNA (% in genome)	1	1	1	1	1
23s_rRNA (% in genome)	1	1	1	1	1
sRNA (% in genome)	15	15	15	15	15

### Comparative genomic analysis of MTB isolates from CTB and PTB

We selected whole genome sequences of 188 PTB isolates from Shanghai from 2009 to 2015 to compare the MTB isolates from different infection sites. These strains had a significantly higher proportion of Lineage 2.2 (177/188, 94.15%) compared to CTB strains. The drug resistance mutation rate in the PTB group (48/188, 25.53%) was significantly higher compared to the CTB group (χ^2^ = 4.286, *p* = 0.031, OR = 2.498, 95%CI: 1.062–5.876) (Yang T. et al., 2018). To eliminate selection bias caused by the potential geographic influence due to the genetic similarity of PTB isolates in this city or by our filter process for low-quality PTB samples, we further compared the lineage distribution and drug resistance rate of CTB strains with PTB strains from Jiangsu or Anhui province in the same period. The genotype distribution showed a similar proportion of Lineage 2.2 (Beijing family), but a much lower drug resistance mutation rate could still be found in CTB strains (Liu et al., 2011; Yao et al., 2020) (Table 3).

**Figure 2:**
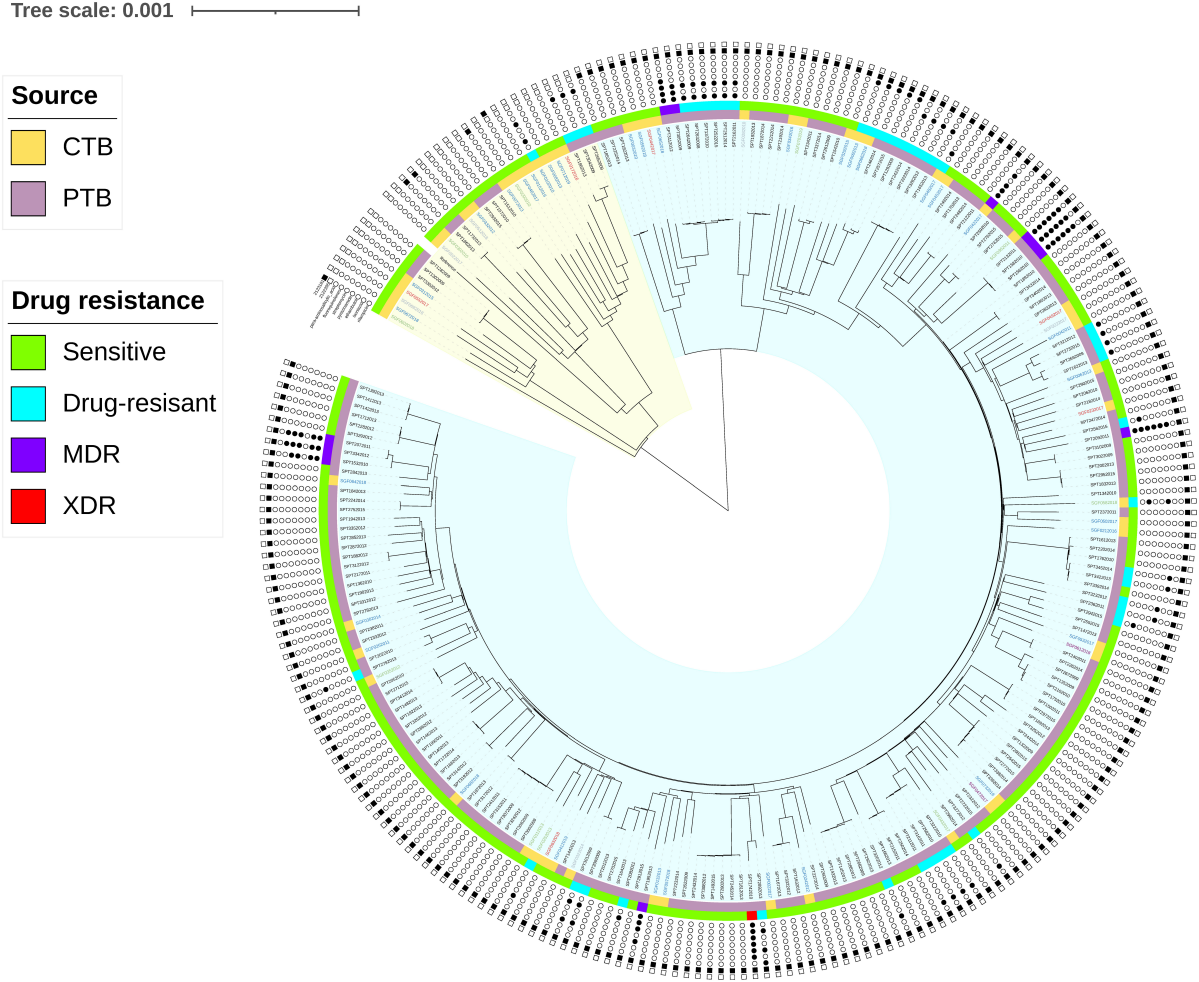
Phylogeography of the 246 MTB isolates (58 CTB and 188 PTB). Strains contained the SNPs on drug targets (INH, RFP, EMB, PNZ, STR, FLQ, and PASA) and loci under convergent evolutionary (Rv1872c (c.757G>A), 2123281; Rv1873 (c.8C>T), 2122395) are marked with a black dot. The branches in the yellow and blue range of the tree belong to Lineage 4 and Lineage 2, respectively. The inner stripes in orange (CTB) and purple (PTB) show the infective location, and the outer stripes in green (sensitive), blue (DR), purple (MDR), and red (XDR) show the drug sensitivity of the strains. MDR was defined as MTB strains with resistance to rifampicin and isoniazid. XDR was defined as MDR strains with resistance to any fluoroquinolone (FQ) and any second-line injectable drug.

In all the CTB isolates, 2,197 SNPs including 1,168 (60.77%) non-synonymous mutations, 734 (38.35%) synonymous SNPs, and 1,738 INDELs (751 insertions and 987 deletions) were detected. The phylogenetic tree constructed on whole genome SNPs between both PTB and CTB isolates showed that the CTB isolates were scattered on the tree. No clade formed by any specific clinical group was observed (Figure 2). SNP diversity among CTB strains ranged from 114 to 1,263, with an average of 654 SNPs. This finding revealed a high degree of genetic heterogeneity, and no transmission incident was detected among the CTB isolates in the study period.

Genome-wide association study (GWAS) is an effective tool in identifying genetic variants related to different clinical forms. Given the small dataset of CTB isolates and the existence of many low-frequency variants, a gene-based burden test was used to find the cumulative effect of these mutations. Out of 178 genes with non-synonymous variants correlated with the two infection forms (*p* < 10^−2^), the following four genes were identified to have strong associations (*p* < 10^−4^): Rv0392c (10.34% of CTB, 15.96% of PTB), Rv2088 (63.79% of CTB, 91.49% of PTB), Rv2331 (0.00% of CTB, 4.26% of PTB), and Rv3829c (5.17% of CTB, 14.89% of PTB). In the homoplastic analysis of the CTB group, we found two missense variations that were supposed to be homoplastic SNPs. They were also detected in PTB strains, namely, Rv1872c (c.757G>A), the coding gene of *lldD2*, had variation in 72.41% of CTB (42/58) and 95.21% of PTB (179/188) and Rv1873 (c.8C>T) was in the subsequent region of Rv1872c, was found in 3.45% of CTB (2/58), and was not detected in PTB.

## Discussion

Among the current nine lineages of the *M. tuberculosis* complex, the Beijing family (Lineage 2.2) and Euro-American lineages (Lineage 4) are the most prevalent lineages in China (Napier et al., 2020). The CTB isolates in this study were also composed of strains of Lineage 2.2 and Lineage 4, which conform to the MTB epidemic status in China. The Beijing family was predominant in both PTB and CTB strains, while Lineage 4 had diverse genotypes in CTB isolates. Lineage 4 highly prevails in the western regions of China. Thus, we suggest that the highly diverse Lineage 4 subtypes in CTB isolates may be caused by the CTB patients coming from multiple regions. The included strains showed scattered distribution in each clade, and no cluster of the same clinical form was found on the phylogenetic tree, which suggests that the isolates of different infection sites were in similar evolutionary positions (Figure 1). Similarly, no significant difference in the accessory genome was found among different CTB types. SMRT sequencing also showed the coincident genomic composition of the four representative isolates and the MTB-type strain. Therefore, we consider that the clinical diversity of CTB has little relationship with the genetic changes in MTB. The lower consistency of CTB isolates is probably associated with the sporadic incidence of CTB (Lin et al., 2020).

Unlike PTB, CTB is generally neglected in many countries. The reason is that CTB is less contagious than PTB. In addition, CTB also usually responds well to first-line anti-tuberculosis therapy (Hill and Sanders, 2017; Zhang et al., 2018). In this study, the drug target mutation rate of CTB isolates was significantly lower than that of PTB isolates, which coincides with the high cure rates of CTB (93.33%). In the comparison of the drug-resistant mutant proportion with PTB isolates from Jiangsu and Anhui provinces, the significantly lower drug-resistant mutant rate showed no relationship with geographical distribution (Liu et al., 2011; Yao et al., 2020). CTB often presents as a chronic infection with mild symptoms, and MTB has few opportunities to spread MTB among patients. Thus, we suggest that the less drug-resistant mutation of these isolates may be due to a long infection time and less transmission or drug treatment experience (Allué-Guardia et al., 2021).

We used a genome-wide association study based on both homoplasy counting and allele counting approaches to find any subtle genetic variations that may cause different phenotypes. Two missense mutations in CTB isolates were detected as homoplastic SNPs. Rv1872c, which is the coding gene of L-lactate dehydrogenase (*lldD2*), is essential for L-lactate reversion as a carbon source *in vitro* (Billig et al., 2017). The knock-out variant failed to utilize L-lactate and barely adapted to the internal environment of infected macrophages. The V253M in the promoter region has been reported with a high probability of positive selection in MTB (Osório et al., 2013). However, a high mutation rate of Rv1872c was also detected in clinical isolates from PTB, which proved that it is not a unique mutation in CTB isolates. Rv1873 is downstream of Rv1872c, and the Rv1873 product reveals limited similarity to various proteins (Garen et al., 2006). The S3L mutant of Rv1873 was detected only in 3.45% (2/58) of CTB isolates and was not found in any PTB isolates, which suggest the possibility of independent evolution in skin infection. Among the 1676 coding genes analyzed in the burden test, four genes with cumulative missense SNP effects were marked as significantly different in the distribution of the PTB and CTB groups. Rv3829c is a probable phytoene dehydrogenase and may play a key role in the transformation of carotenoids from colorless to colored (Rose et al., 2013). Rv0392c is an NADH dehydrogenase coding gene with a membrane-bound domain and participates in energy metabolism. The product was found with a higher abundance in multidrug-resistant strains and could cause antibiotic resistance if overexpressed (Phong et al., 2015). Rv2088 (*PknJ*) is a transmembrane serine/threonine protein kinase involved in the regulation of pyruvate kinase A and essential for survival under stress conditions (Singh et al., 2014). Rv2331 is a putative nitrate reductase with pH sensitivity activated by the virulence regulator PhoP (Bansal et al., 2017). Notably, all these genes are engaged in mycobacterium metabolism. In particular, Rv3829c and Rv0392c encode dehydrogenase genes. In this study, strains with mutations in Rv0392c and Rv3829c were found in 36 and 31 samples, respectively. The two mutants overlapped in more than 50% of the strains, and most of the missense variants led to decreased protein stability in prediction (8/10 in Rv0392c, 8/8 in Rv3829c) (Capriotti et al., 2005). Meanwhile, significantly more drug-resistant mutations were found in strains with mutations in the two genes (Rv0392c, 15/36, 41.67%, χ^2^ = 9.0576, *p* = 0.003; Rv3829c, 15/31, 41.67%, χ^2^ = 13.844, *p* < 0.001). Thus, we suggest that the two genes synergistically exert influence and maybe the fitness cost of antibiotic-resistant mutations and lead to lower adaptability to skin infection.

This study provided the first genomic datasets of isolates from CTB and identified the molecular characteristics of MTB isolated from CTB in southeastern China. The results showed similar genomic composition and genotyping results in strains from the four major types of CTB and PTB, which suggested that the various cutaneous infection types could barely be affected by the genetic differences of MTB. It also suggested the rare but general risk of cutaneous infection in tuberculosis patients. However, we found lower drug mutation rates of CTB isolates and differences in SNPs of metabolism-related genes among the two groups. The lower genetic variation rate in drug-related genes evidenced the low antibiotic resistance in clinical practice and supported 6-month drug therapy (2HRZE + 4HR) recommended by the WHO for CTB. The metabolic genes found by comparative genomic methods and their relationships with drug resistance provide a reference for future studies. The major limitation of this study is a small dataset of CTB isolates compared with PTB cases involved, which may weaken the effect of comparative genome analysis and fail to draw definitive conclusions. This impact could be diminished by including more isolates in future studies. Another limitation is that other potential genomic factors that may contribute to the development of CTB are ignored, which could be improved by enhancing the overall sequence quality of the samples.

## Data availability statement

The datasets presented in this study can be found in online repositories. The names of the repository/repositories and accession number(s) can be found below: https://www.ncbi.nlm.nih.gov/, PRJNA820632.

## Ethics statement

The study involving human participants was reviewed and approved by the ethics committee of Hospital of Skin disease, Institute of Dermatology, Chinese Academy of Medical Sciences and Peking Union Medical College. Written informed consent to participate in this study was obtained from all participants.

## Author contributions

HSW contributed to the conception of the study. WYZ, JSX, LW, YQC, SYL, and CP contributed to data collection. YMM, HQJ, and YS performed the experiment. YMM, JYS, and TL contributed to data analysis. YMM wrote the original draft. HSW and TL contributed to review and editing. All authors contributed to the article and approved the submitted version.

## Supplementary material

Supplementary Table 1 Sequencing information of the 58 CTB isolates.
